# Silicon Mitigates Salinity Stress by Regulating the Physiology, Antioxidant Enzyme Activities, and Protein Expression in* Capsicum annuum* ‘Bugwang'

**DOI:** 10.1155/2016/3076357

**Published:** 2016-03-20

**Authors:** Abinaya Manivannan, Prabhakaran Soundararajan, Sowbiya Muneer, Chung Ho Ko, Byoung Ryong Jeong

**Affiliations:** ^1^Division of Applied Life Science (BK21 Plus), Graduate School, Gyeongsang National University, Jinju 660-701, Republic of Korea; ^2^Institute of Agriculture and Life Science, Gyeongsang National University, Jinju 660-701, Republic of Korea; ^3^Research Institute of Life Science, Gyeongsang National University, Jinju 660-701, Republic of Korea

## Abstract

Silicon- (Si-) induced salinity stress resistance was demonstrated at physiological and proteomic levels in* Capsicum annuum* for the first time. Seedlings of* C. annuum* were hydroponically treated with NaCl (50 mM) with or without Si (1.8 mM) for 15 days. The results illustrated that saline conditions significantly reduced plant growth and biomass and photosynthetic parameters and increased the electrolyte leakage potential, lipid peroxidation, and hydrogen peroxide level. However, supplementation of Si allowed the plants to recover from salinity stress by improving their physiology and photosynthesis. During salinity stress, Si prevented oxidative damage by increasing the activities of antioxidant enzymes. Furthermore, Si supplementation recovered the nutrient imbalance that had occurred during salinity stress. Additionally, proteomic analysis by two-dimensional gel electrophoresis (2DE) followed by matrix-assisted laser desorption/ionization time-of-flight mass spectrometry (MALDI-TOF-MS) revealed that Si treatment upregulated the accumulation of proteins involved in several metabolic processes, particularly those associated with nucleotide binding and transferase activity. Moreover, Si modulated the expression of vital proteins involved in ubiquitin-mediated nucleosome pathway and carbohydrate metabolism. Overall, the results illustrate that Si application induced resistance against salinity stress in* C. annuum* by regulating the physiology, antioxidant metabolism, and protein expression.

## 1. Introduction

Salinity is a major abiotic stress that limits the growth and yield of agricultural and horticultural crops worldwide. Primarily, salinity hampers the osmotic balance in plants by affecting the electrochemical gradients and vascular transportation of solutes [1]. In higher plants, salt stress leads to several physiological and metabolic modulations such as retardation of photosynthesis, ion toxicity, oxidative burst, and nutrient imbalance [2–5]. In addition, higher accumulation of Na^+^ and Cl^−^ ions during saline conditions hinders the uptake of essential nutrients [6]. Furthermore, salinity accelerates the production of harmful reactive oxygen species (ROS) that cause oxidative damage to proteins, lipids, and nucleic acids by affecting normal cellular metabolism [7]. Hence, an alternative strategy of silicon (Si) supplementation to overcome the negative effects of salinity in plants can be considered as a valuable approach.

Silicon is the second most abundant element in the Earth's crust, covering 27.70% of the lithosphere. The essential roles of Si in plant systems have been extensively studied by numerous plant biologists for several years, but by definition Si is considered as a “quasi-essential” or nonessential element for plants, because most plant species can complete their life cycle without it [8]. However, there are several hypotheses concerning the physiological functions of Si in monocots and dicots. Under abiotic stress like salinity, Si application resulted in the alleviation of stress and enhancement of plant growth [9, 10]. During salt stress, the apoplastic transport of Na^+^ and Cl^−^ ions was decreased by Si deposition [11, 12]. According to Zhu and Gong [13], the mechanisms behind silicon-mediated alleviation of salt stress include the following aspects: (a) maintenance of optimal water content; (b) enhancement of photosynthesis and curbing transpiration rate; (c) limiting oxidative stress by alleviating ion toxicity; and (d) biosynthetic regulation of solutes and plant hormones. In line with other researchers, Al-aghabary et al. [14] observed increased activities of antioxidant enzymes and enhanced photochemical efficiency of PSII under salt stress.

Although the beneficial effects of Si against abiotic stresses are evident from previous reports, to date there is a lack of understanding of the molecular regulation of Si-mediated stress tolerance. In order to gain a deeper insight into Si induced salt tolerance in pepper plants, proteomic analysis based on two-dimensional gel electrophoresis- (2DE-) mass spectrometry (MS) has been employed in the present study. Moreover, proteomic strategies are considered the best molecular approach to study the dynamics of proteins, particularly the response of Si in a stressed environment [15–18]. Therefore, to our knowledge, for the first time, the current study has attempted to investigate the effect of Si on the growth, physiology, antioxidant enzyme activities, nutrient content, and protein expression in* C. annuum* under salinity stress.

## 2. Materials and Methods

### 2.1. Plant Material and Treatments

Seeds of* Capsicum annuum* “Bugwang” were surface sterilized with 0.5% sodium hypochlorite for 10 min followed by washing in double distilled water 4 times. After sterilization, the seeds were sown on seed germination trays containing commercial Tosilee medium (Shian Precision Co., Jinju, Republic of Korea). After one week, the seedlings were subjected to salinity and Si treatments. For the treatments, seedlings were transplanted to magenta boxes containing 300 mL of nutrient solution formulated according to Soundararajan et al. [9]. Each magenta box consisted of four plants, and silicon was supplemented in the form of potassium silicate (K_2_SiO_3_). The excess potassium introduced by the K_2_SiO_3_ was deducted from potassium nitrate and the nitrate loss was balanced by the addition of nitric acid. Salinity stress was provided by the addition of sodium chloride (NaCl) to the nutrient solution. The pH of the nutrient solution was adjusted to 5.70. Totally, the experiment consisted of four treatments such as control (basal nutrients without NaCl or Si), Si alone (1.8 mM), NaCl alone (50 mM), and Si + NaCl (Si-1.8 mM; NaCl-50 mM). All the treatments were arranged in a randomized block design with three replicates. The experiment was conducted in a glass house at Gyeongsang National University, Jinju, Republic of Korea, under normal daylight conditions with day/night set temperature of 27/19°C and relative humidity (RH) 60–70%.

### 2.2. Measurement of Growth and Photosynthesis

After 15 days of treatment, biomass and photosynthetic parameters were measured. The net photosynthesis rate (*P*_*n*_), stomatal conductance (*G*_*s*_), and transpiration rate (*T*_*r*_) were measured using a LI-6400 portable photosynthetic measurement system (Li-COR, Inc, Lincoln, NE, USA). For the microscopic observation of stomata, the epidermal layer of photosynthetically active second leaves in all the treatments were peeled and stained with 0.01% toluidine blue O. After staining the stomatal structures were observed under a light microscope in 20x magnification and photographed using Nikon Y-TV55.

### 2.3. Determination of Oxidative Stress Biomarkers

The level of oxidative stress was determined by estimation of electrolyte leakage potential (ELP), malondialdehyde content (MDA), and hydrogen peroxide levels.

For ELP measurements, the leaf discs (0.5 cm) were washed with distilled water, immersed in 10 mL distilled water for 22 h, and autoclaved for 120 min at 90°C. Electrical conductivity (EC) was measured before and after autoclaving to determine the electrolyte leakage. The ELP % was calculated according to Campos et al. [18].

The lipid peroxidation level in the leaves was estimated based on the MDA content according to Zhu et al. [7]. Briefly, 0.1 g of leaf samples was homogenized in trichloroacetic acid (TCA) solution (0.1%, 5 mL) and centrifuged at 18,000 ×g for 15 min. The supernatant (0.5 mL) was added to 5 mL of 0.5% thiobarbituric acid (TBA) solution prepared in 20% TCA. The reaction mixture was incubated in a hot water bath (95°C) for 30 min and the reaction was terminated by keeping the mixture on ice. After 5 min, the samples were centrifuged for 5 min at 10,000 ×g and the absorbance was determined at 532 nm. By subtracting the nonspecific values at 600 nm, the MDA content was calculated using the extinction coefficient (155 mM^−1^ cm^−1^).

The spectrophotometric determination of H_2_O_2_ content was carried out according to Christou et al. [19]. Briefly, 0.1 g of leaf sample was homogenized in 1 mL of 0.1% TCA and centrifuged at 10,000 ×g for 15 min. Subsequently, 0.5 mL of supernatant was mixed with 10 mM phosphate buffer (0.5 mL, pH 7.0) and 1 mL potassium iodide (1 M). The mixture was incubated at 25°C for 30 min and the absorbance was measured at 390 nm. The H_2_O_2_ content was determined from a standard calibration curve.

### 2.4. Estimation of Antioxidant Enzymes Activity

For the analysis of antioxidant enzymes, 0.1 g of tissue was homogenized in 50 mM phosphate buffer (pH 7.0) containing 1 mM EDTA, 0.05% triton X, and 1 mM polyvinylpyrrolidone (PVP). Then the homogenate was centrifuged at 10,000 ×g for 20 min at 4°C and the supernatant was used for determination of antioxidant enzymes activity. The activity of SOD was assayed by following the nitro blue tetrazolium (NBT) inhibition method of Giannopolitis and Ries [20]. GPX activity was measured based on the amount of enzyme required for the formation of tetraguaiacol per minute, according to Shah et al. [21]. CAT enzyme activity was determined according to the method of Cakmak and Marschner [22]. APX activity was estimated by following the protocol of Nakano and Asada [23]. The total protein content of the samples was estimated according to the Bradford method [24] using a bovine serum albumin (BSA) standard curve.

### 2.5. Two-Dimensional Polyacrylamide Gel Electrophoresis (2D-PAGE)

#### 2.5.1. Protein Extraction

For 2D-PAGE analysis, protein extraction was carried out by following the procedure of Muneer et al. [25]. In detail, leaf tissue (0.1 g) was homogenized in liquid nitrogen using a prechilled pestle and mortar. The proteins were extracted with a commercial protein extraction kit (Bio-Rad, Hercules, CA, USA) according to the instructions provided by the manufacturer. For total protein isolation, about 2 mL of extraction buffer [8 M urea, 4% 3-[(3-cholamidopropyl) dimethylammonio]-1-propanesulfonate (CHAPS), 40 mM Tris, 0.2% bio-lyte (*pI* 3–10)] was mixed with lyophilized (0.1 g) leaf tissue. The homogenate was vortexed and sonicated with an ultrasonic probe to disrupt any interfering substances such as genomic DNA and phenolics. After sonication, the samples were centrifuged for 30 min at 4°C and the supernatant was transferred to new Eppendorf tubes. The resultant supernatant was employed for isoelectric focusing after protein quantification with the Bradford method using bovine serum albumin (BSA) standard curve.

#### 2.5.2. Two-Dimensional Gel Electrophoresis and Silver Staining

A total of 70 *μ*g of dissolved protein sample was separated by 2DE in the first dimension by isoelectric focusing on a 7 cm IPG strip (*pI* 4–7) (GE Healthcare, UK) and the second dimension by SDS-PAGE on a Protean II unit (Bio-Rad Hercules, USA), according to methods given by Muneer et al. [25]. The samples were rehydrated for 12 h (with 125 *μ*L rehydration buffer containing 70 *μ*g proteins) before focusing. For the first dimension, the rehydrated strips were focused at 20°C with 50 *μ*A current per strip using a four-step program: step and hold -300 V for 30 min, gradient, -1000 V for 30 min, gradient, -5000 V for 1 h 30 min, and final step and hold 1-2 h until the final voltage reached 10000 V. The focused strips were equilibrated twice for 15 min in 10 mg·mL^−1^ DTT and then in 40 mg·mL^−1^ iodoacetamide prepared in equilibration buffer containing 50 mM Tris-HCl (pH 8.8), 6 M urea, 30% (v/v) glycerol, and 2% (w/v) SDS. After equilibration, strips were attached to the second dimension gel (12.5%) with 0.5% low melting point agarose sealing solution. Electrophoresis was done at a constant voltage of 80 V for 4 h until the bromophenol dye front reached the end of the gel. The protein spots in the analytical gels were stained using a silver staining method [26].

#### 2.5.3. Image Acquisition and Data Analysis

Three replicate gels from each treatment were used for image acquisition and data analysis. Spot detection, spot measurement, background subtraction, and spot matching were performed using Progenesis SameSpots*™* 2D software (ver. 4.1, Nonlinear Dynamics, Newcastle, UK) in automatic spot detection mode to review the annotations of spots statistically using one-way ANOVA analysis (*n* = 3, *P* < 0.05) at a 95% confidence level. The differentially expressed proteins spots were identified as spots showing more than a twofold change in expression on comparison with control.

#### 2.5.4. In-Gel Digestion of Protein Spots

The differentially expressed protein spots were excised manually from the gels [27] and washed with distilled water three times. The protein spots were chopped and destained with 30 mM potassium ferricyanide and 100 mM sodium thiosulphate pentahydrate (1 : 1) by incubating at room temperature for 30 min. The destaining reagent was removed and the gel particles were treated with 100 *μ*L of 50 mM NH_4_HCO_3_ for 5 min and dehydrated in 30 *μ*L of acetonitrile for 5 min. After dehydration, the gel was covered with 100 *μ*L reduction solution (10 mM dithiothreitol in 50 mM NH_4_HCO_3_) and incubated for 45 min at 56°C. After the removal of reduction solution, 100 *μ*L of alkylation solution (100 mM iodoacetamide in 50 mM NH_4_HCO_3_) was added and incubated at 25°C in the dark for 30 min. Finally, the gel pieces were washed with 30 *μ*L of 50 mM NH_4_HCO_3_ for 5 min and dehydrated with 30 *μ*L of acetonitrile for 10 min. After drying using a vacuum centrifuge, the gel pieces were rehydrated in 5 to 10 *μ*L of 25 mM NH_4_HCO_3_ containing 5 ng·*μ*L^−1^ trypsin (Promega, Madison, WI, USA) at 37°C for 30 min. After incubation, the excess trypsin solution was replaced with 5 to 10 *μ*L of 25 mM NH_4_HCO_3_ and digestion was carried out for a minimum of 16 h at 37°C. The digested peptides were subsequently pooled, vacuum dried, and mixed with 3 *μ*L of sample solution (50% acetonitrile and 0.1% trifluoroacetic acid).

#### 2.5.5. Peptide Identification

For protein identification, the tryptic digested peptide mixtures were targeted onto a MALDI-TOF-MS plate and analyzed by a Voyager-DE STR mass spectrometer (Applied Biosystems, Franklin Lakes, NJ, USA), equipped with delay ion extraction. Mass spectra were obtained over a mass range of 800–3500 Da. Homology search was executed by matching the experimental results with both theoretical digests and sequence information from public protein databases using Mascot software (http://www.matrixscience.com/). Search parameters employed were as follows: carbamidomethyl cysteine as a fixed modification and oxidation of methionine as a variable modification, one missed cleavage site, and peptide mass tolerance of ±100 ppm. NCBI-nr database with the taxonomy Viridiplantae (green plants) was employed to identify regions of similarity between sequences. The protein score employed was −10*∗*log⁡(*P*), where *P* is the probability that the observed match is a random event. The spot identities were submitted to a gene ontology (GO) retriever (http://www.agbase.msstate.edu/) and the resulted annotations were summarized based on the GOSlim set using a GOSlim Viewer. The hierarchical clusters of the treatments were generated with the Cluster 3.0 program followed by a heat map analysis with the TreeView tool.

### 2.6. Statistical Analysis

To find the statistical significance between treatments data collected were subjected to analysis of variance (ANOVA) followed by Duncan's multiple range test at *P* ≤ 0.05 and *F*-test using the SAS program (Statistical Analysis System, V. 6.12, Cary, USA). All sets of data were represented as the means of three replicates each.

## 3. Results and Discussion

### 3.1. Analysis of Growth, Biomass, and Photosynthetic Parameters

**Figure 1:**
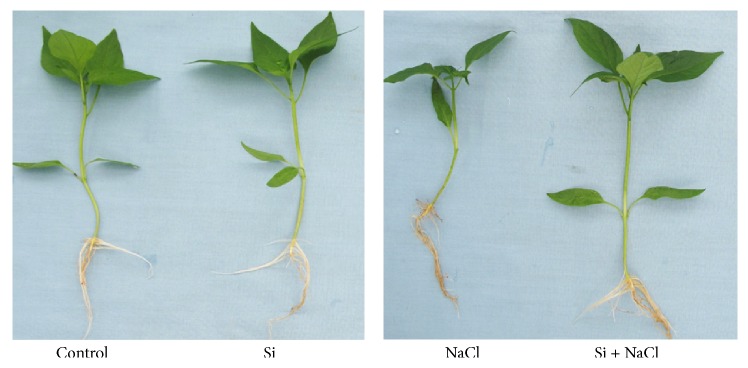
Effects of Si supplementation and salinity stress on the growth of* C. annuum*.

**Figure 2:**
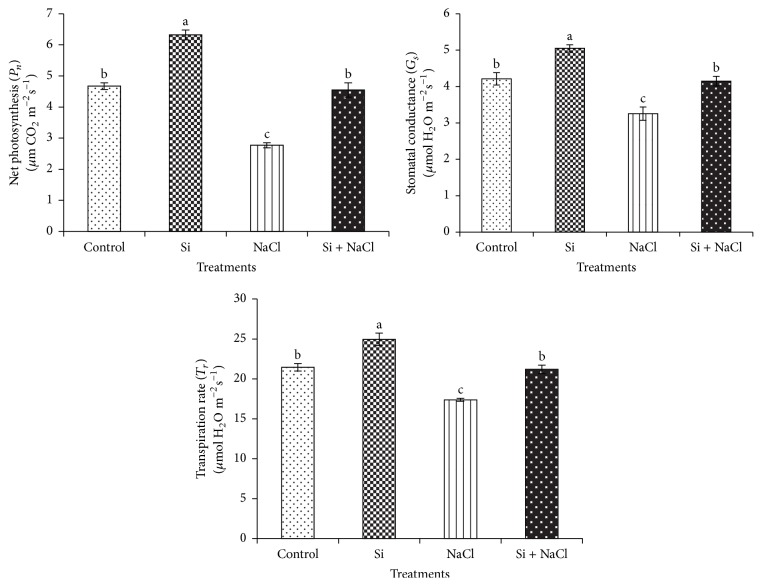
Effects of Si nutrition and salinity stress on photosynthetic parameters of* C. annuum*. Different letters in one measurement indicate statistically significant differences at *P* ≤ 0.05 using Duncan's multiple range test.

**Figure 3:**
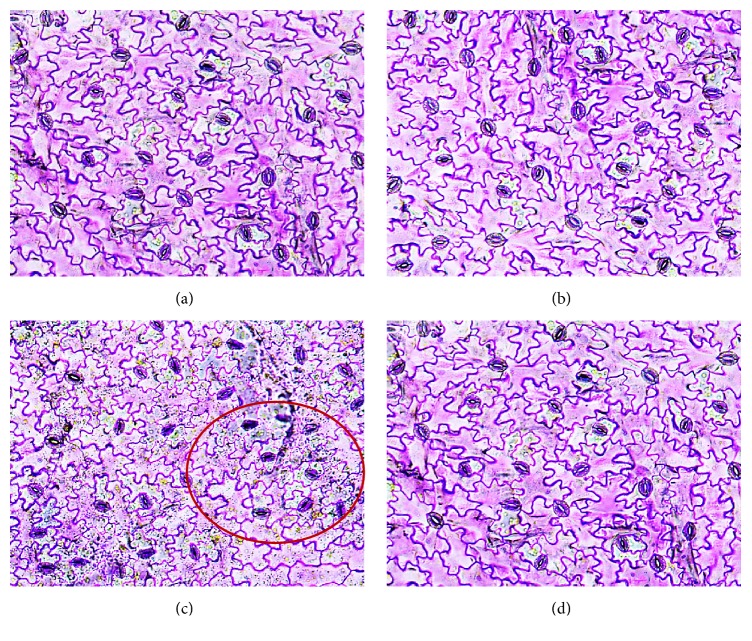
Effects of Si supplementation and salinity stress on stomata of* C. annuum* in 20x magnification. (a) Control, (b) Si, (c) NaCl treatment with closed stomata that was denoted by the red circle, and (d) Si + NaCl treatment.

Hydroponically supplemented Si significantly increased growth and alleviated salinity stress in* C. annuum* (Figure 1). Growth parameters and tissue Si content measured after 15 days of salinity and Si treatments are shown in Table 1. The deleterious effects of salt stress on the growth and biomass of* Capsicum* were significantly mitigated by Si supplementation. The uptake of Si by* C. annuum* was 1.24 ± 0.6 mg·g^−1^ DW in Si treatment whereas during salinity stress the tissue Si content was increased to 1.61 ± 0.31 mg·g^−1^ DW. Besides the Si treatments, negligible amount of Si was identified in control and NaCl treatments. This could have been acquired during seed germination, because the seedlings were irrigated with normal tap water during seed germination and grown in Tosilee medium, a substrate with a negligible amount of Si. Moreover, salinity treatment significantly decreased the net photosynthesis rate, stomatal conductance, and transpiration rate by 41.3%, 23.8%, and 19.1%, respectively. However, Si treatment alleviated the deleterious effect of salt on the photosynthetic parameters (Figure 2). Stomatal observations illustrated that in NaCl treatment the majority of the stomata were observed in closed state, whereas plants in the Si alone and Si-treated NaCl conditions contained prominent open stomata consistent with control plants (Figure 3). In line with previous reports [13, 14], salt stress impaired the physiology and morphology of* Capsicum* plants by manifesting a water imbalance which led to a reduction in growth and biomass. This damage was recovered by application of Si in* Capsicum*. Si was previously reported to improve growth, biomass, and photosynthesis by imparting mechanical strength to the plants under stress conditions [11–14].

### 3.2. Evaluation of Oxidative Stress Biomarkers

**Figure 4:**
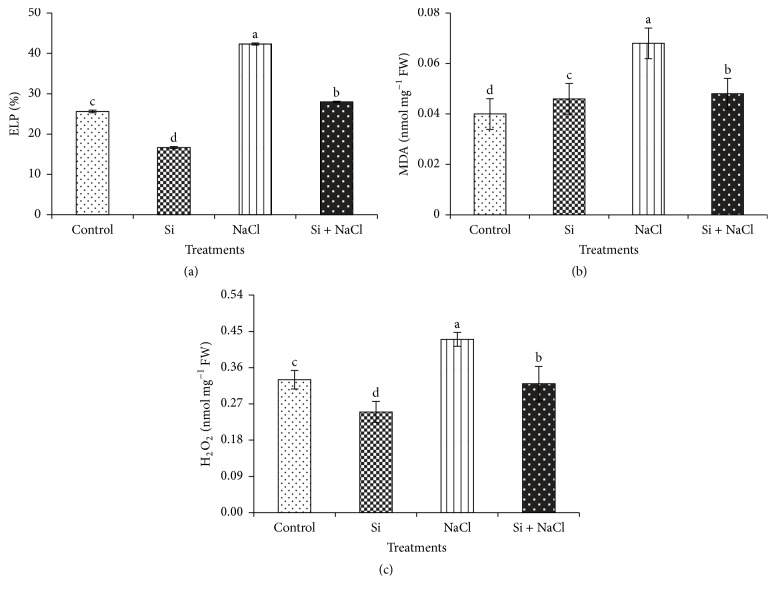
Si treatment and salinity stress on biochemical stress markers. (a) Electrolyte leakage potential (ELP); (b) malondialdehyde content (MDA); and (c) hydrogen peroxide content (H_2_O_2_). Data are the mean ± SE from three replicates. Different letters in one measurement indicate statistically significant differences at *P* ≤ 0.05 using Duncan's multiple range test.

The membrane potential and oxidative burst induced by salinity treatment in* Capsicum* was assessed by electrolyte leakage potential (ELP), malondialdehyde (MDA) content, and hydrogen peroxide levels, respectively (Figure 4). Significant increase in ELP levels in salt stress treatment by 38.1% illustrated the NaCl induced cell membrane damage, which in turn was reduced by 33.3% upon Si supplementation (Figure 4(a)). Because of the imbalance in electrolyte leakage potential, there was an increase in lipid peroxidation (Figure 4(b)) and hydrogen peroxide content (Figure 4(c)) in salinity treatments. However, the addition of Si mitigated the oxidative damage by decreasing the MDA content by 29.4% and H_2_O_2_ content by 25.6%. In general, higher NaCl concentration causes dysfunction of cell membranes that leads to the excess permeability of ions and electrolytes, which tend to increase the oxidative burst in cells [7, 9]. Si-mediated alleviation of oxidative damage by strengthening the structural integrity of cell membranes, particularly during salinity stress, has been reported in several plants [7].

### 3.3. Estimation of Antioxidant Enzyme Activities

**Figure 5:**
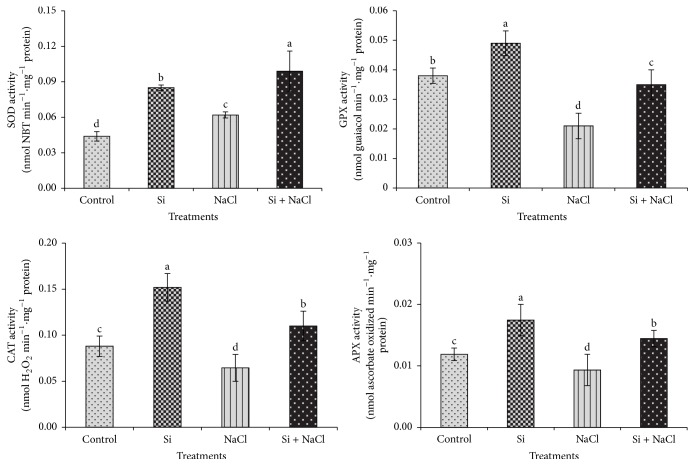
Modulation of activities of antioxidant enzymes upon Si supplementation and salinity stress. Different letters in one measurement indicate statistically significant differences at *P* ≤ 0.05 using Duncan's multiple range test.

Under salt stress, Si significantly increased the activity of antioxidant enzymes such as superoxide dismutase (SOD), guaiacol peroxidase (GPX), catalase (CAT), and ascorbate peroxidase (APX) (Figure 5). The enhancement of antioxidant enzyme activities by Si under salt stress to protect the plant from oxidative stress has been considered as one of the primary mechanisms of salt stress alleviation induced upon Si supplementation [9, 11]. However, our experimental plants in NaCl treatment displayed the lowest antioxidant enzyme activities, denoting a perturbation of antioxidant enzyme metabolism. In contrast, salinity induced an enormous production of harmful H_2_O_2_. Higher accumulation of H_2_O_2_ during salt stress impaired the defense of antioxidant metabolism, leading to an imbalance between the production and elimination of ROS. However, the supplementation of Si controlled the generation of H_2_O_2_ and restored the balance between ROS production and its scavenging by enhancing the activities of antioxidant enzymes [7]. Briefly, Si treatment increased the activity of SOD under salinity stress. SOD plays an important role as the primary line of defense by catalyzing the dismutation of detrimental superoxide radical to hydrogen peroxide, which is further detoxified by GPX, CAT, and APX at the cost of different substrates [7]. In the Si added NaCl treatment the detoxification of ROS by GPX, an antioxidant enzyme which is important for the metabolism of polyphenols, has been enhanced [28]. Similarly, the CAT enzyme which is a universal oxidoreductase responsible for the fine regulation of H_2_O_2_ for the signaling process [29] has been regulated by the Si supplementation. In addition, the APX enzyme involved in the reduction of H_2_O_2_ via ascorbate-glutathione cycle by utilizing ascorbate as the substrate electron donor [30] has been improved by Si. Overall, the activities of antioxidant enzymes were enhanced with the addition of Si in both normal and salinity stress conditions. Our results are concordant with previous reports illustrating Si-mediated modulation of antioxidant enzymes that contribute to the abiotic stress tolerance [7].

### 3.4. Analysis of Capsicum Leaf Proteome under Salinity Stress and Si Treatment

#### 3.4.1. Investigation of 2DE Protein Profiles

**Figure 6:**
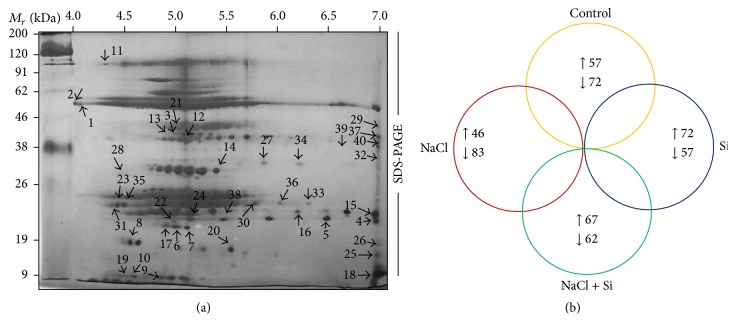
(a) Master 2DE gel representing the differentially expressed protein spots in pI range 4–7 resolved in 7 cm IPG strip. (b) Venn diagram illustrating the differential expression of* C. annuum* leaf proteins under Si and salinity treatments. Numbers correspond to the protein spots present in 2DE patterns of control, Si, NaCl, and Si + NaCl treatments. The upward and downward arrows denote increased or decreased protein expression under four treatments.

**Figure 7:**
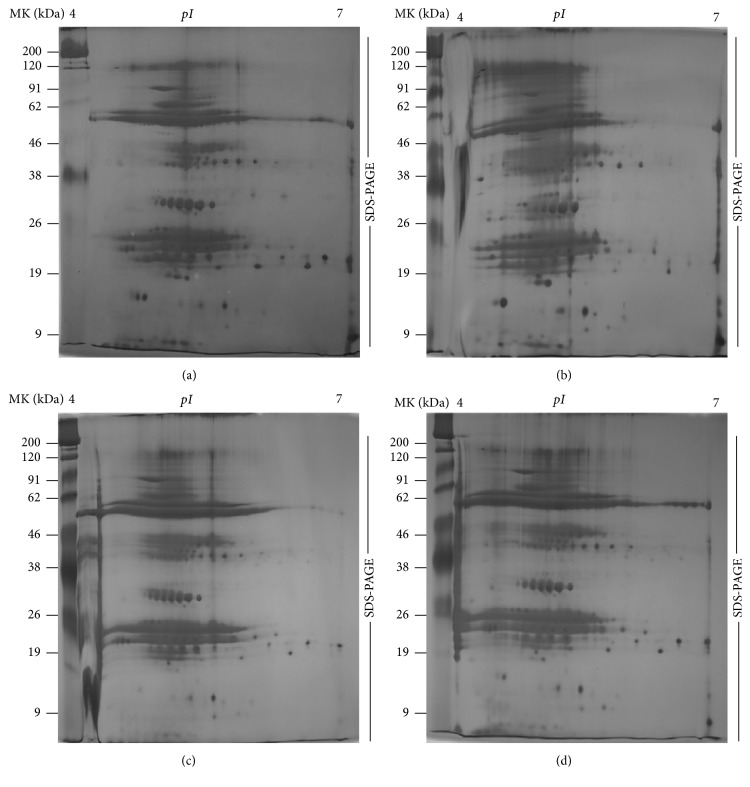
2DE gels displaying the differential expression profiles of proteins across the treatments; (a) control, (b) Si, (c) NaCl, and (d) Si + NaCl treatments.

In addition to the above-mentioned physiological and biochemical factors, we utilized proteomics tools to investigate the molecular effects of Si on improving the resistance against salt stress. High resolution 2DE patterns with protein spots resolved in a pI range of 4–10 are shown in Figure 6(a). The comparative analysis of 2DE gels analyzed by Progenesis SameSpots TotalLab (Newcastle, UK) revealed that 245 protein spots were reproducibly resolved amongst the three replicates. Amongst the resolved spots, 129 protein spots were differentially expressed with more than 2.0-fold change compared to control. Moreover, Si supplementation without NaCl stress significantly upregulated 72 spots, and 57 proteins were highly expressed in the control treatments. Significantly, salinity stress downregulated 83 spots and upregulated 46 spots. Interestingly, Si supplementation during NaCl stress increased the regulation of 67 protein spots (Figure 6(b)). Several proteins spots that were detected only in control and Si treatments were absent in the treatment with NaCl alone (Figure 7). The decrease in protein expression in NaCl treatment can be due to the progressive reduction of metabolic pathways associated with signal transduction and gene regulation involved in protein synthesis [31, 32]. In addition, the excessive production of ROS, which leads to incorrect folding or assembly of proteins, can be associated with consequent protein degradation in salt-stressed* Capsicum* [33].

#### 3.4.2. Peptide Identification Using Matrix-Assisted Laser Desorption/Ionization Time-of-Flight Mass Spectrometry (MALDI-TOF MS)

From the 129 spots that were analyzed, proteins from 40 spots were identified using MALDI-TOF MS.  Table 2 shows the list of identified proteins along with the corresponding spot ID, nominal mass, theoretical and calculated pI, accession number, MASCOT score, and percentage of sequence coverage. The percentages of sequence coverage of proteins identified in* C. annuum* were in the range of 14–100%. The expression levels of the identified protein spots have been listed in supplementary Figure S1 (see Supplementary Material available online at http://dx.doi.org/10.1155/2016/3076357). Si supplementation without salinity stress upregulated the proteins involved in several metabolic process. Adenylosuccinate synthase (spot 1), an important enzyme in purine metabolism, was upregulated by Si. Nucleotide metabolism plays a vital role throughout the growth and development of plants [34]. The increased expression of purine metabolism-related proteins by Si may be attributed to the enhancement of growth and biomass in* C. annuum*. In addition, Si treatment enhanced the expression of E3 ubiquitin ligase (spot 2), which catalyzes the third major step in the ubiquitination process. The enzyme E3 ligase significantly influences several developmental processes such as photo-morphogenesis, floral development, senescence, and circadian rhythm of plants [35]. Similarly, Si increased the expression of carbon fixation and photosynthesis-related proteins such as RuBisCo (spot 5) and oxygen evolving enhancer protein (spot 12), respectively. Spots 5 and 12 were repressed by salinity, representing the degradation of photosynthesis and energy-related metabolic processes, as reported in several plant species [15–18].

Si treatment caused the accumulation of a nucleoporin-like protein (spot 7). Nucleoporins are involved in several vital roles, especially plant disease resistance and hormone signaling [36]. Probable calcium binding protein-CML17 (spot 9) and mitochondrial calcium uniporter regulatory subunit MCUb-like isoform (spot 19) were found to be increased by Si treatment. In general, calcium is considered a universal secondary messenger with well-defined roles in several cellular responses, and calcium binding proteins also act against several stresses [37, 38]. Thus, the enrichment of calcium transportation-related proteins by Si could benefit the plants during environmental stresses. Apart from the above-mentioned proteins, Si supplementation also enhanced the expression of RNA polymerase II transcription subunit 11 (spot 16), ribosomal protein L16 (spot 17), and a resistance protein candidate (spot 24). Salinity stress resulted in the downregulation of several proteins; however it led to the upregulation of zinc finger protein-160 (spot 3). Zinc finger proteins have been widely known to control stomatal aperture movements to avoid excess water loss during salt and drought stresses [39]. Therefore, an increase in the expression levels of zinc finger protein can be interpreted as a stress tolerance response activated in* C. annuum* to combat ROS imbalance and water loss. Similarly, spot 37 corresponding to glyceraldehyde-3-phosphate, a vital enzyme in several metabolic processes including glycolysis, was upregulated in NaCl treatment. According to Jeong et al. [40], the expression of glyceraldehyde-3-phosphate was significantly increased by abiotic stresses like salinity. Moreover, NaCl induced the accumulation of molybdopterin synthase catalytic subunit (spot 13), a key enzyme that catalyzes the synthesis of a molybdenum cofactor in the abscisic acid (ABA) biosynthetic pathway [41]. Thus, the increase in spot 13 emphasizes the activation of ABA biosynthesis, which is a prominent stress response observed in several plants [42]. Salinity also upregulated *β*-keto acyl reductase (spot 25), a rate limiting enzyme involved in fatty acid metabolism [43], uncharacterized protein LOC104086136 isoform X2 (spot 33), reverse transcriptase (spot 34), eukaryotic translation initiation factor 3 subunit D (spot 35), minichromosome maintenance 5 protein (spot 36), Ras-related protein RABH1b-like isoform X3 (spot 39), and F-box/kelch-repeat protein (spot 40). Furthermore, the combination of Si and NaCl treatments resulted in enhanced accumulation of DEMETER-like protein-2 (spot 11), a key regulator of DNA methylation, particularly under a stressful environment [44]. MADS-box transcription factor 26 isoform X2 (spot 14) was enhanced by the combined application of Si and NaCl. MADS-box transcription factors are associated with wide range of functions, particularly in growth and development [44]. Importantly, under salt stress conditions, addition of Si upregulated cullin 1D (spot 28) and F-box protein 8-like (spot 29), which are vital proteins involved in the ubiquitin-proteasome pathway. These proteins are involved in the regulation of signal transduction, light response, floral development, self-incompatibility, epigenetic regulation, and stress resistance [45]. Moreover, the increase in ubiquitination-related proteins in Si-treated NaCl conditions implies that, in order to remove the stress-induced defective proteins, the ubiquitin-cascade-mediated protein degradation might be induced. In addition, the accumulation of phosphoglycerate kinase (spot 23), ATP-synthase CF1*α* subunit (spot 27), disease resistance protein RPS2 (spot 15), and double-stranded RNA binding protein 2 (spot 30) associated with major metabolic processes was increased in Si + NaCl treatment. Thus, regulation of the above-mentioned proteins involved in the growth, development, and stress resistance processes gives detailed information about the improved physiology, photosynthesis, antioxidant metabolism, and nutrient balance induced by Si, particularly under salt stress conditions.

#### 3.4.3. Gene Ontology (GO) and Clustering Analysis

**Figure 8:**
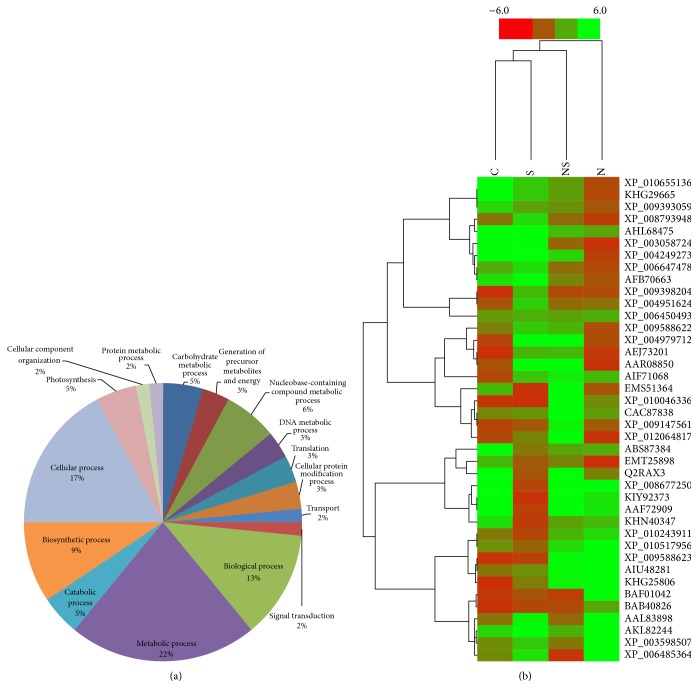
(a) Gene ontology analysis of the identified proteins from* C. annuum* leaves. (b) Hierarchical clustering analysis of the differentially expressed proteins in response to Si supplementation and salt stress. The dendrogram of the spot clusters with the relative expression values of individual proteins is displayed as a heat map. All quantitative information is transmitted using a color scale ranging from red for the downregulation to green for the upregulation. Each row is representative of a single spot and each column indicates the treatment (C: Control; S: Si; NS: Si + NaCl; N: NaCl).

The biological processes of the identified proteins analyzed by gene ontology (GO) are illustrated in Figure 8(a). The GO results indicated that a large proportion of the differentially regulated proteins was involved in metabolic processes (22%), followed by cellular processes (17%), biological processes (13%), biosynthetic processes (9%), nucleobase containing compound metabolic processes (6%), photosynthesis (5%), carbohydrate metabolism (5%), catabolic processes (5%), cellular protein modification (3%), DNA metabolism (3%), translation (3%), generation of precursor metabolites and energy (3%), signal transduction (2%), transport (2%), cellular component organization (2%), and protein metabolic processes (2%). Of the 22% of proteins involved in metabolic processes, most are involved in phosphorylation, oxidation-reduction process, glycolysis and carbon fixation, reductive pentose phosphate cycle, DNA replication, methylation, transcription, translation, photorespiration, and AMP biosynthesis.

In addition, majority of the differentially expressed proteins were involved in nucleotide binding (23%), particularly ATP and GTP binding, followed by transferase activity (17%), catalytic activity (14%), kinase activity (11%), protein binding (6%), DNA binding (5%), chromatin binding (3%), RNA binding (3%), structure molecule activity (3%), sequence-specific DNA binding transcription factor activity (3%), translation factor activity (3%), and hydrolase activity (3%) (Figure S2A). Since the proteins were isolated from leaf tissues, the cellular components (Figure S2B) of the proteins were largely delineated to plastids (26%). Other locations of the proteins were traced to intracellular (16%), cellular component (16%), cell (11%), nucleus (11%), thylakoid (5%), ribosome (5%), membrane (5%), and cytoplasm (5%). The result of hierarchical clustering analysis of all the treatments is represented as a heat map in Figure 8(b). Column 3 in the heat map representing the Si + NaCl treatment illustrated that Si treatment induced the upregulation of proteins against salt stress. In addition, the downregulation of proteins in NaCl treatment was identified, in accordance with alfalfa [15].

Taken together, the results illustrate that Si significantly mitigated salinity stress in* Capsicum* by maintaining the physiology, biochemical parameters, and antioxidant enzyme metabolism and regulated the expression of leaf proteins.

## 4. Conclusions

In conclusion, the present study reveals that Si protects* Capsicum* from salinity stress by alleviating oxidative stress and enhancing growth by regulating photosynthesis, integral nutrient management, and antioxidant enzyme metabolism. Supplemented Si induced the expression of proteins involved in photosynthesis, cellular metabolism, and stress resistance to mitigate the salt stress. Thus, our results indicate that supplementation of Si plays an indispensable role in the regulation of leaf proteins and alleviation of oxidative damage caused by salinity stress in* C. annuum*.

## Supplementary Material

Supplementary Figure S1: The expression levels of identified proteins revealed the salinity stress-mediated depletion of important proteins. However, the exogenous application of Si significantly improved the expression of proteins by regulating the complex physiological mechanisms. Thus, the graphical representation of the spot volumes provides an additional insight into the Si-induced protein regulation.

## Figures and Tables

**Table 1:** Growth parameters of *Capsicum annuum* affected by salinity stress and Si supplementation.

Si (mM)	NaCl (mM)	Shoot length (cm)	Shoot diameter (mm)	Root length (cm)	Number of roots	Fresh wt. (g)	Dry wt. (g)	Si content (mg·g^−1^ DW)
0	0	8.82 ± 0.18c^z^	1.58 ± 0.12c	7.04 ± 0.02c	22.40 ± 0.04e	1.22 ± 0.11b	0.15 ± 0.10a	0.47 ± 0.18c
	50	6.80 ± 0.10d	1.12 ± 0.16d	13.56 ± 0.15a	23.20 ± 0.21e	0.91 ± 0.04c	0.09 ± 0.08bc	0.48 ± 0.07c
1.8	0	11.70 ± 0.15b	2.07 ± 0.05a	13.40 ± 0.02a	40.40 ± 0.10c	2.15 ± 0.10a	0.18 ± 0.11a	1.24 ± 0.6b
	50	13.40 ± 0.08a	1.81 ± 0.07b	9.50 ± 0.03b	43.20 ± 0.03b	2.11 ± 0.12a	0.13 ± 0.05b	1.61 ± 0.31a

^z^Different letters in one measurement indicate statically significant difference at *P* ≤ 0.05 by Duncan multiple range test.

Data are represented as mean ± SE from three replicates.

**Table 2:** List of differentially expressed proteins identified by MALDI-TOF/MS in *C. annuum* leaves.

Spot number	Accession number	Nominal mass (*M*_*r*_)	Theoretical *pI/*experimental *pI*	Protein identification	Species	Sequence coverage (%)	MASCOT Score
1	XP_004249273	55408	7.5/4.2	Adenylosuccinate synthetase, chloroplastic	*Solanum lycopersicum*	25	51
2	XP_008793948	44084	8.2/4.1	E3 ubiquitin-protein ligase PUB23-like	*Phoenix dactylifera*	20	56
3	XP_010243911	57603	5.0/4.8	Zinc finger protein 160-like	*Nelumbo nucifera*	17	53
4	XP_010655136	78992	6.4/6.4	Vacuolar protein sorting-associated protein 53 A isoform X2	*Vitis vinifera*	16	66
5	AHL68475	27519	6.7/5.0	Ribulose-1,5-bisphosphate carboxylase/oxygenase, partial (chloroplast)	*Androcymbium cf. capense Chacon 20*	41	47
6	KHG29665	13451	11.4/5.0	tRNA-specific-2-thiouridylase mnmA	*Gossypium arboreum*	50	42
7	XP_009393059	42588	6.1/5.1	GDP-mannose 3,5-epimerase 2-like	*Musa acuminata *subsp. *malaccensis*	32	46
8	XP_003058724	59677	9.1/4.6	Nucleoporin-like protein	*Micromonas pusilla *CCMP1545	33	43
9	XP_006647478	17489	4.2/4.8	Probable calcium-binding protein CML17-like	*Oryza brachyantha*	46	63
10	EMT25898	18876	8.5/4.6	Ribulose bisphosphate carboxylase small chain PWS4, chloroplastic	*Aegilops tauschii*	80	44
11	EMS51364	17984	8.9/4.3	DEMETER-like protein 2	*Triticum urartu*	50	69
12	XP_009398204	24720	9.5/5.1	Oxygen-evolving enhancer protein 3-1, chloroplastic-like	*Musa acuminata *subsp. *malaccensis*	52	64
13	XP_010517956	20595	6.5/4.8	Molybdopterin synthase catalytic subunit-like	*Camelina sativa*	80	62
14	XP_008677250	28739	8.8/5.4	MADS-box transcription factor 26 isoform X2	*Zea mays*	42	51
15	XP_010046336	55906	5.3/7.0	Disease resistance protein RPS2-like	*Eucalyptus grandis*	19	45
16	XP_004951624	13105	5.6/6.2	Mediator of RNA polymerase II transcription subunit 11-like	*Setaria italica*	59	48
17	AFB70663	15211	11.8/4.7	Ribosomal protein L16, partial (chloroplastic)	*Weingartia kargliana*	63	52
18	AEJ73201	5179	6.2/7.0	CIN-like protein, partial	*Nandina domestica*	53	43
19	XP_009588622	33538	9.2/4.4	Mitochondrial calcium uniporter regulatory subunit MCUb-like isoform	*Nicotiana tomentosiformis*	26	46
20	XP_004979712	107060	6.3/5.5	Putative disease resistance RPP13-like protein 3-like	*Setaria italica*	14	52
21	ABS87384	56157	5.0/4.9	Pyruvate kinase	*Lactuca sativa*	20	56
22	BAF01042	57010	8.7/4.8	Receptor-like protein kinase	*Arabidopsis thaliana*	41	50
23	KIY92373	29267	8.7/4.4	Phosphoglycerate kinase, partial	*Monoraphidium neglectum*	51	54
24	AAR08850	4803	9.4/5.2	Resistance protein candidate	*Vitis amurensis*	100	55
25	AAL83898	10928	11.6/6.9	Beta-keto acyl reductase	*Zea mays*	87	45
26	AAF72909	18001	5.4/7.0	Resistance gene analog protein	*Medicago sativa*	56	38
27	AIF71068	21906	8.6/5.8	ATP synthase CF1 alpha subunit, partial (chloroplast)	*Actiniopteris semiflabellata*	49	44
28	CAC87838	26888	5.0/4.5	Cullin 1D	*Nicotiana tabacum*	25	53
29	XP_009147561	34297	9.4/7.0	F-box only protein 8-like	*Brassica rapa*	33	53
30	XP_012064817	54515	8.7/5.7	Double-stranded RNA-binding protein 2	*Jatropha curcas*	14	57
31	XP_006450493	45127	6.0/4.4	Hypothetical protein CICLE_v10010442mg, partial	*Citrus clementina*	19	43
32	Q2RAX3	52023	7.1/7.0	CBL-interacting protein kinase 33	*Oryza sativa *japonica group	13	47
33	XP_009588623	22039	8.3/6.4	Uncharacterized protein LOC104086136 isoform X2	*Nicotiana tomentosiformis*	39	54
34	BAB40826	16893	7.9/6.3	Reverse transcriptase	*Zea mays*	30	46
35	KHG25806	41699	8.9/4.4	Eukaryotic translation initiation factor 3 subunit D	*Gossypium arboreum*	26	50
36	AIU48281	79429	7.5/6.0	Minichromosome maintenance 5 protein, partial	*Illicium henryi*	27	50
37	AKL82244	11565	8.1/7.0	Glyceraldehyde 3-phosphate dehydrogenase, partial	*Rosa soulieana*	84	52
38	KHN40347	11468	5.6/5.4	Putative caffeoyl-CoA O-methyltransferase	*Glycine soja*	37	63
39	XP_006485364	19263	8.9/6.6	Ras-related protein RABH1b-like isoform X3	*Citrus sinensis*	31	47
40	XP_003598507	43923	6.8/7.0	F-box/kelch-repeat protein	*Medicago truncatula*	22	47
