# Supplementary material for: Silicon Mitigates Salinity Stress by Regulating the Physiology, Antioxidant Enzyme Activities, and Protein Expression in Capsicum annuum ‘Bugwang'
Source: Biomed Res Int. 2016 Mar 20;2016:3076357. doi: 10.1155/2016/3076357 (PMC4818800; doi:10.1155/2016/3076357)
Supplement: Supplementary file 1 — Supplementary Figure S1: The expression levels of identified proteins revealed the salinity stress-mediated depletion of important proteins. However, the exogenous application of Si significantly improved the expression of proteins by regulating the complex physiological mechanisms. Thus, the graphical representation of the spot volumes provides an additional insight into the Si-induced protein regulation. [file 3076357.f1.pdf]

Supplementary figures:

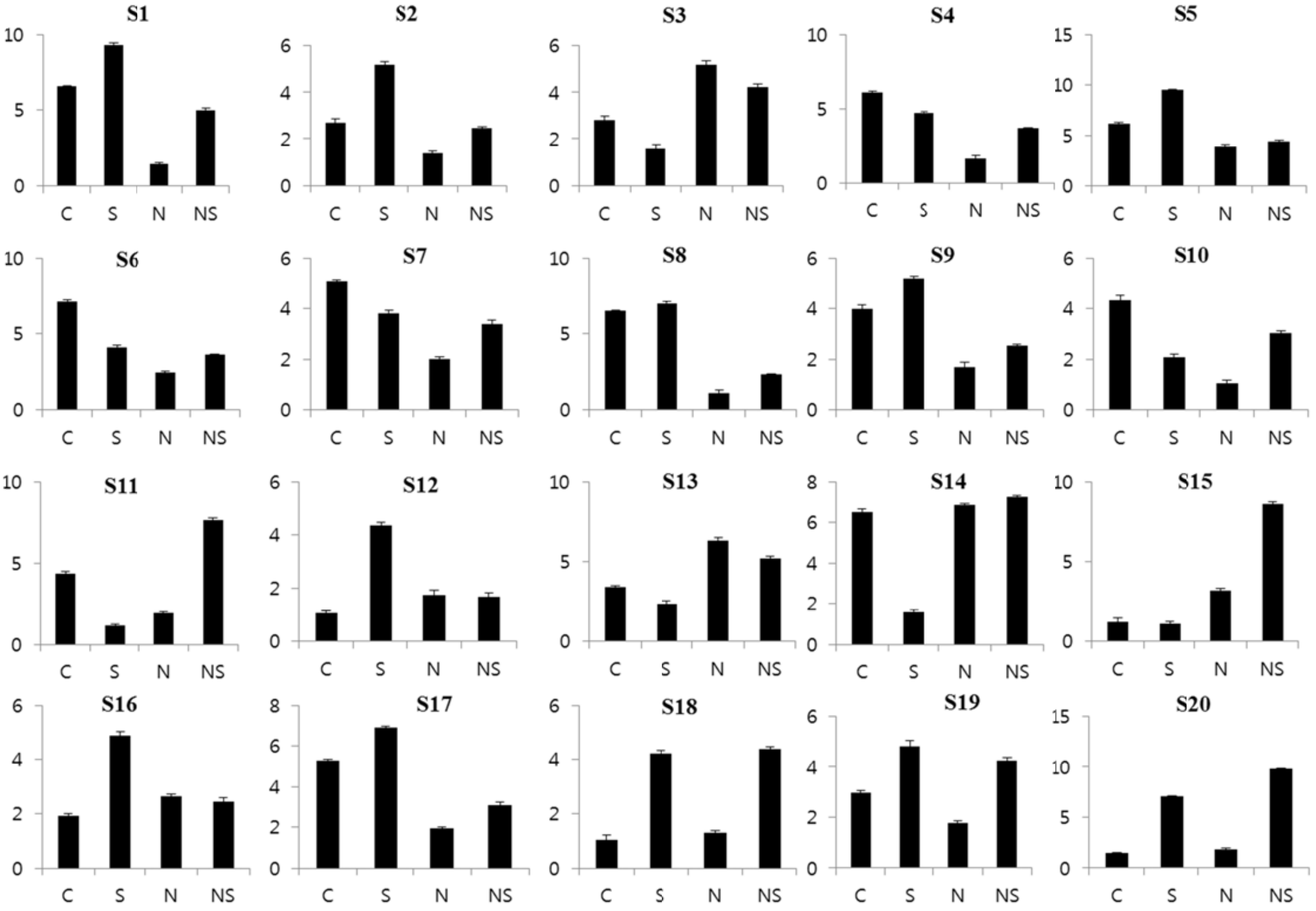

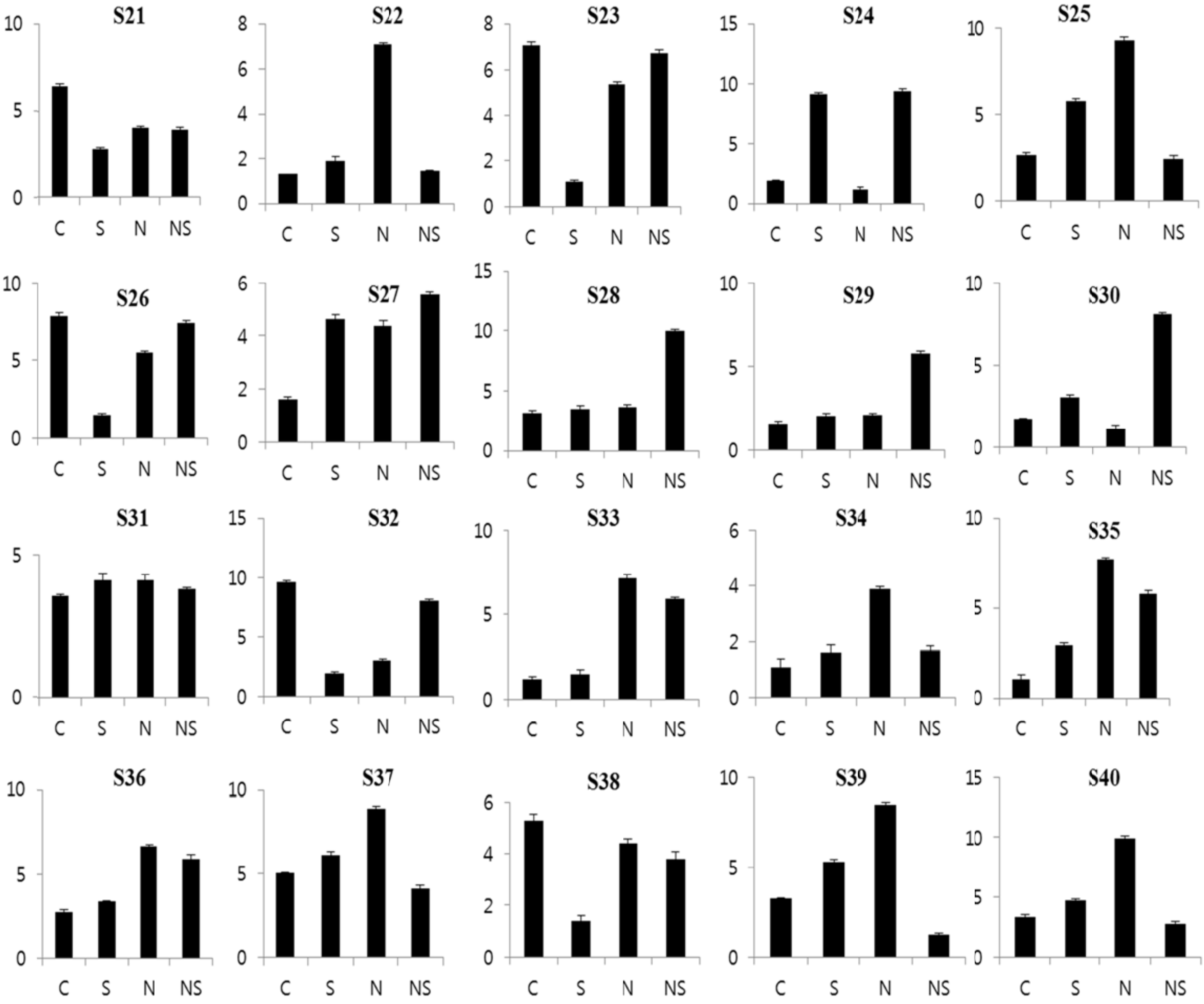

Figure S1. Graphical representation of spot volume mean of proteins. Spot volume mean, presented for 40 differentially regulated proteins upon Si application and salt stress (C-Control; S- Si; NS- Si + NaCl; N- NaCl).

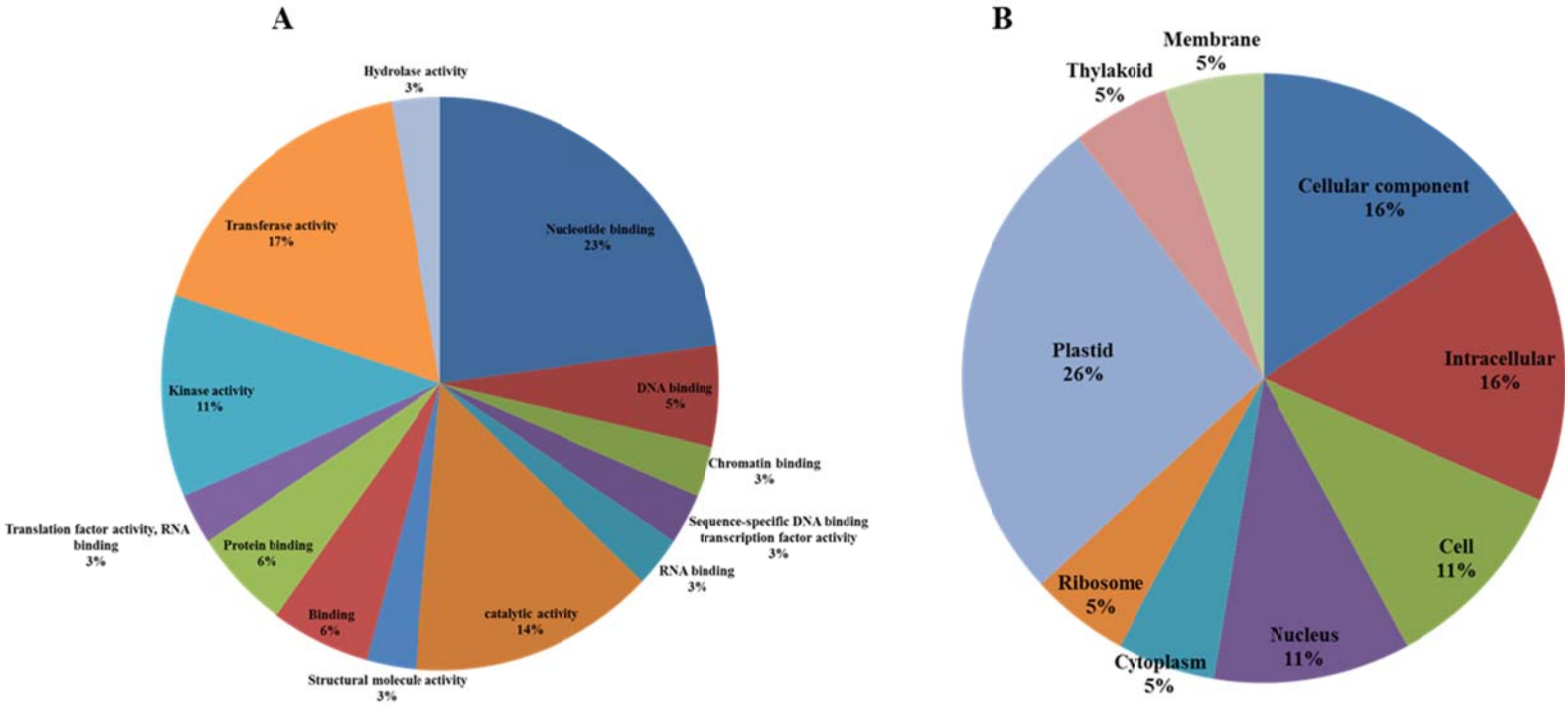

Figure S2. Gene ontology results of *C. annuum* proteins. (**A**) Putative functions. (**B**) Cellular component.
